# Does the Optimal Dietary Methionine to Cysteine Ratio in Diets for Growing Chickens Respond to High Inclusion Rates of Insect Meal from *Hermetia illucens*?

**DOI:** 10.3390/ani8110187

**Published:** 2018-10-23

**Authors:** Anne Brede, Christian Wecke, Frank Liebert

**Affiliations:** Department of Animal Sciences, Division of Animal Nutrition Physiology, Georg-August-University of Goettingen, 37077 Goettingen, Germany; anne.brede@agr.uni-goettingen.de (A.B.); cwecke@gwdg.de (C.W.)

**Keywords:** growing chickens, sulfur containing amino acids, methionine, cysteine, amino acid digestibility, protein quality, amino acid efficiency, insect meal, *Hermetia illucens*

## Abstract

**Simple Summary:**

Currently, several alternative protein sources are under investigation for replacing soybean meal in poultry diets. One alternative is larvae meal of the black soldier fly (*Hermetia illucens)* with a specific sulfur amino acid composition. The larvae meal is limiting in sulfur amino acids supply and provides a wide methionine:cysteine ratio of 61:39. Currently, it is not known whether the insect meal has an impact on the optimal ratio of methionine to cysteine in broiler chicken diets. The methionine:cysteine ratio significantly influences animal growth and welfare. Both methionine and cysteine excess and deficiency can lead to an impaired feed intake, growth, and feed efficiency. Therefore, the aim of this study was to investigate whether the optimal methionine:cysteine ratio is modulated when a high inclusion rate of partly defatted *Hermetia illucens* meal is applied. The results show that a methionine:cysteine ratio of 50:50 yields superior growth and dietary protein quality. It can be concluded that the insect meal under study is a promising alternative protein source without modulating the optimal methionine:cysteine ratio in broiler chicken diets.

**Abstract:**

The dietary methionine:cysteine (Met:Cys) ratio (MCR) is an important factor influencing the optimal growth of chickens. Therefore, this study aimed to contribute to the assessment of the optimal dietary MCR in diets with the complete replacement of soybean meal (SBM) by a partly defatted larvae meal of *Hermetia illucens* (HM). A growth study with 240 male meat-type chickens (Ross 308) was conducted, also assessing the body nutrient deposition both at the end of the starter (day 21) and the grower (day 35) period. Birds were fed experimental diets based on wheat, maize, and insect meal (23%/21% HM in starter/grower diets). Sulfur amino acids were created as the limiting AA in diets with graded MCR (40:60; 45:55; 50:50; 55:45; 60:40). The control diet contained SBM instead of HM with a MCR of 50:50. The current results based on growth parameters, dietary protein quality, and Met efficiency data gave support to the previous assumption of an ideal MCR of 50:50, which was also valid in diets with a high proportion of insect meal. The lowest MCR of 40:60 led to significantly impaired feed intake and growth of the birds, while the response to the highest MCR (60:40) was moderate.

## 1. Introduction

The sulfur containing amino acids (SAA) methionine (Met) and cysteine (Cys) play important roles in cell metabolism [1]. For example, Met initiates the protein synthesis in all eukaryotic cells [1,2]. Furthermore, Met is a methyl and sulfur donor and an important factor for antibody response [3,4,5,6]. Cys is directly involved in body sulfur transport processes, active sulfate biosynthesis, and the synthesis of, e.g., taurine, coenzyme A, and glutathione [7,8,9]. Met is mainly found in the muscles [10,11], while the feathers are characterized by a high Cys content [12]. This underlines the importance of these amino acids (AA) for growth and feather development in meat-type chickens. A dietary deficiency of Met or Cys leads to a significant decline in feed intake, and as a consequence, impairs growth and feed efficiency, as well as yields alterations in carcass composition [10,11,13,14,15]. Similar effects on feed intake, growth, and feed efficiency have also been observed due to an oversupply of Met or Cys [16,17,18,19,20,21].

However, most of the feedstuffs used in poultry nutrition do not contain a sufficient quantity of Met and Cys. In consequence, SAA are often the most limiting factor for feed protein quality in frequently used chicken diets [5,9,22,23,24]. In order to compensate the SAA-deficiency, broiler chicken diets are usually supplemented with either DL-Met, L-Met or a Met-hydroxy-analogue [25]. Aviagen [26,27] recommends 1.08–0.82% SAA and 0.56–0.42% Met in practical diets for Arbor Acres, Ross 308 and Ross 708 broilers depending on the age of the birds providing a Met:Cys ratio (MCR) of 52:48.

However, literature data reporting the optimal Met:Cys ratio (MCR) in broiler diets differ markedly. While the National Research Council (NRC) [28] recommended a Cys percentage of 43 or 47% of the SAA for the starter and grower diet, Baker et al. [8], Powell et al. [29], and Khan et al. [30] proposed a MCR of 50:50 in broiler chicken diets. Wheeler and Latshaw [31] stated that diets for growing chickens should maximally contain 54% and 38–43% Cys in the starter and grower period, respectively. However, the results of Farke et al. [32] and Sünder et al. [33] indicated a sparing effect for Met at a Cys proportion >50% of the total SAA content. One important influencing factor is that the metabolic degradation of the indispensable AA Met may yield Cys [1,34,35,36]. Consequently, an appropriate amount of Cys leads to a decrease in cystathionine synthase and, therefore, increases the availability of Met [37]. However, the close metabolic link between Met and Cys makes it more difficult to assess both the animals´ requirement for total SAA and the optimal dietary MCR. In order to minimize the metabolic degradation of the indispensable Met, it is not only important to know the total SAA requirement, but also the optimal dietary ratio between Met and Cys. The methodology used in AA requirement studies is only valid for the indispensable Met, but determining under which metabolic conditions Met degradation is found to be minimized could provide more information about the optimal dietary supply of Cys.

Currently, conventional feeds for growing chickens usually contain soybean meal (SBM) as the main protein source. However, several alternative protein sources are under investigation to lower the contribution of imported proteins to animal feeds. One possible alternative could be insect meal from Black Soldier Fly, *Hermetia illucens* (HM), larvae, which is characterized by a high protein content and a well-balanced AA profile [38,39,40,41,42,43,44,45]. Recent studies have demonstrated that the replacement of SBM by HM from 5% up to 100% is possible in AA balanced diets for both laying hens [41] and growing chickens [44,45,46,47,48,49,50,51]. However, according to the specific AA composition high inclusion rates of insect meals may alter the MCR in chicken diets and it remains unknown whether they affect the efficiency of dietary Met or Cys.

Therefore, the aim of this study was to evaluate the dietary Met efficiency at graded MCR in mixed feeds for growing chickens with a high inclusion rate of HM, as an alternative protein source that completely replaces SBM.

## 2. Materials and Methods

The experiment was conducted at the facilities of the Division Animal Nutrition Physiology, Department of Animal Sciences, Georg-August-University of Goettingen. Investigations were approved by the Animal Welfare Committee of Lower Saxony, Germany (AZ 17A119).

### 2.1. Birds and Housing

To begin, 240 male day-old chickens (Ross 308) were purchased from a commercial hatchery and kept on wood shavings in a floor pen for the first 24 h while receiving a standard starter diet. The next day, the birds were individually weighed and allotted to 48 experimental pens in order to yield a similar average body weight (BW; BW_start_ = 55 g) per pen at the start of the experiment. Each pen (≥1.05 m^2^) was equipped with an individual feeder and an automatic drinking system (five birds per pen). Birds were bedded on wood shavings. The growth study was divided into a starter (day 1–21) and a grower period (day 22–35). Housing temperature was 32–26 °C in the starter and 25–22 °C in the grower period. Monochromatic light was provided for 23 h per day.

### 2.2. Diets and Feeding

The diets were based on wheat, maize, and SBM or insect meal. The AA content of the main ingredients is shown in Table 1.

The AA content of the experimental diets (Table 2) was set according to the currently recommended [52] ideal amino acid ratio (IAAR), making exceptions for Met and Cys. The ratio of total SAA to lysine was fixed at 0.50 in order to ensure the limiting position of SAA in each of the diets under study. This was the precondition for evaluating the dietary AA efficiency based on the application of the “Goettingen approach” (see Section 2.5). Diets A–E contained 23.00/22.28% (starter/grower period) partly defatted HM with graded MCR from 40:60 to 60:40 (see Table 1). The insect meal was provided by a commercial producer (Hermetia Futtermittel GbR, Baruth/Mark, Germany) and contained 60.8% crude protein (CP), 14.1% crude lipids (CL), and 7.5% crude ash (CA) on a dry matter (DM) base. For further information see also Neumann et al. [46,47] and Velten et al. [48,49]. SBM was utilized as the reference protein source in diet F at a fixed MCR of 50:50. Grower diets were manufactured by dilution of starter diets with wheat starch in order to ensure a constant ratio between the protein sources and a consistent protein quality in the diets. In addition, as an indigestible marker, titanium dioxide was included in grower diets for assessing the apparent precaecal protein and AA digestibility by application of the marker technique.

The pelleted feed was offered ad libitum, and feeders and the drinking system were checked twice a day and cleaned if needed. The analyzed nutrient composition of the diets is shown in Table 3.

### 2.3. Chemical Analysis

Feed ingredients as well as final diets, chyme, and animal bodies were analyzed according to the national standards of the Association of German Agricultural Analytic and Research Institutes (VDLUFA) [54]. Chyme samples were freeze-dried for 48 h (Model α 1-4^®^, Christ GmbH, Osterode am Harz, Germany) before analysis. Whole bodies were homogenized without defrosting using a band saw (Model FK 22, Bizerba SE & Co. KG, Balingen, Germany) and a mincing machine (Model EM 82 VL-S, Edertal Elektromotoren GmbH & Co. KG, Wabern, Germany). A representative sample of the body homogenate was freeze-dried before analysis. This time-consuming procedure was applied to prevent body SAA losses during usual autoclaving processes [24,55] before further homogenization. The N content was quantified by the Dumas method (TruMac^®^, Leco Instrument GmbH, Moenchengladbach, Germany) and CP was calculated with a factor of 6.25. AA analysis was conducted using ion-exchange chromatography (Biochrom^®^ 30, Biochrom Ltd., Cambridge, UK) following acid hydrolysis with or without an oxidation step for the quantitative determination of SAA. CL content was analyzed following HCl hydrolysis of the feed samples. CL content of the animal bodies was calculated by difference (CL = 100-CP-CA) and expressed as percent of DM.

### 2.4. Growth, Feed Efficiency, and Digestibility Data

Individual bird BW and feed intake per pen were monitored weekly in order to calculate daily body weight gain (BWG), dry matter intake (DMI), and feed conversion ratio (FCR). Additionally, 20 birds per diet were chosen for assessing apparent precaecal digestibility (apD). Therefore, pooled precaecal samples (*n* = 5) were collected from the last 2/3 between Meckel´s diverticulum and 2 cm before the ileo-caecal junction after slaughtering on day 36 and were immediately frozen and stored at −20 °C until further analysis. *apD* was calculated as follows:

(1) $$apD\;\left(\%\right)=100-100\times \frac{\left(marker_{feed}\times nutrient_{chyme}\right)}{\left(marker_{chyme}\times nutrient_{feed}\right)}$$

where: *marker_feed_* = concentration of the marker in the feed (% DM), *marker_chyme_* = concentration of the marker in the chyme (% DM), *nutrient_chyme_* = concentration of the *nutrient* (CP, AA) in the chyme (% DM), and *nutrient_feed_* = concentration of the *nutrient* (CP, AA) in the feed (% DM).

### 2.5. Body N deposition, Dietary Protein Quality, and Met Efficiency Parameters

Following a 24 h feed deprivation period, both at the end of the starter (day 21) and grower period (day 35), one representative bird per pen was chosen for whole body N analysis. The selected birds were euthanized via carbon dioxide exposure according to animal welfare regulations and subsequently frozen (−20 °C) until further sample preparation. Based on the comparative slaughter technique, average daily N deposition (*ND*) was calculated using the difference between the analyzed final quantity of body N and initial body N for the starter, grower, and total growth period. Data for the initial quantity of body N were taken from an earlier study with the same genotype [56]. Calculated mean daily *ND* values were related to the corresponding average metabolic BW (mg N/BW_kg_^0.67^).

According to earlier reports [30,57,58,59,60,61,62,63,64] the “Goettingen approach” was applied as follows:

(2) $$NR=NR_{max}T\;\left(1-e^{-NI\times b}\right)$$

(3) $$ND=NR_{max}T\;\left(1-e^{-NI\times b}\right)-NMR$$

(4) $$b=\left[lnNR_{max}T-ln\left(NR_{max}T-NR\right)\right]:NI$$

where: *NR* = daily N retention (mg/BW_kg_^0.67^), *NR_max_T* = daily theoretical maximum of the N retention (mg/BW_kg_^0.67^), *ND* = daily N deposition (mg/BW_kg_^0.67^), *NMR* = daily N maintenance requirement (mg/BW_kg_^0.67^), *NI* = daily N intake (mg/BW_kg_^0.67^), *b* = slope of the N retention curve (indicating the feed protein quality independent of *NI*), and *e* = base number of the natural logarithm (*ln*).

The model parameters for daily *NMR* (240 mg/BW_kg_^0.67^) and *NR_max_T* (4240, 3440, and 3840 mgN/BW_kg_^0.67^/d as applied for starter, grower, and the whole growth periods, respectively) were taken from earlier experiments with the same genotype [62].

Due to the fact that traditional protein quality parameters were not independent of the level of realized protein intake [65,66,67], a standardization of protein intake was conducted by the “Goettingen approach” according to earlier reports [46,47,48,49,60,64,66,67]:

(5) $$NPU_{std.}\left(\%\right)=NR_{max}T\times \frac{\left(1-e^{-b\times NI_{std.}}\right)}{NI_{std.}}\times 100$$

The standardized N intake as applied was 3300 mg/BW_kg_^0.67^ (starter period), 3000 mg/BW_kg_^0.67^ (grower period), and 3200 mg/BW_kg_^0.67^ (total growth period). The model parameter *b* as derived from the exponential function (Equation (3)) is a prerequisite for applying this standardization procedure.

According to Samadi and Liebert [68], the model parameter *b* is closely related to the concentration of the limiting AA (*c*). The quotient *bc*^−1^ describes the slope of this linear relationship and indicates the efficiency of utilizing the limiting AA in the diet [58,66]:

(6) $$bc^{-1}=\frac{b}{c}$$

where: *b* = slope of the N retention curve (indicating the feed protein quality independent of *NI*) and *c* = concentration of the limiting AA in the feed protein (g/16 gN).

### 2.6. Body Protein and Body Fat Deposition

The protein and fat deposition were determined by computing the difference between the analyzed nutrient contents in the whole body of birds at the end and the start of each growth period, respectively. Data for the initial quantity of body protein and body fat were taken from an earlier study with the same genotype [56].

### 2.7. Statistical Analysis

Statistical analyses were conducted with SPSS software package (IBM SPSS Statistics, Version 24.0). One-way analysis of variance (ANOVA) tests were performed to compare means and standard deviations of all data. Dependent on the variance homogeneity (evaluated by Levene-Test), identification of significant differences (*p* ≤ 0.05) was carried out making use of the Games-Howell test and Tukey post-hoc test, respectively.

## 3. Results

### 3.1. Growth and Feed Efficiency

The zoo-technical results (Table 4) demonstrated that in the starter period diet C (HM 50:50) and diet F (SBM 50:50) yielded both superior final body weight (FBW) and daily body weight gain (BWG), but did not significantly differ from diet D (HM 55:45). The response with diet C was significant as compared to the diets with a lower MCR (A,B), but did not differ significantly from diet E (HM 60:40). Significant effects were seen for daily dry matter intake (DMI), which was consistently below diet F (*p* < 0.05), but highest with diet C between SBM substituted diets. Consequently, the feed conversion ratio (FCR) was impaired with diet F, but the effect was insignificant. The best FCR was found in diet C (HM 50:50) with only numerous differences to diets with a higher MCR (D,E) and the SBM diet F. Diet A (HM 40:60) yielded the lowest DMI, and as a consequence, the lowest FBW (*p* < 0.05). In general, modulating the MCR significantly affected zoo-technical data in the starter period. 

Accordingly, the results of the grower period follow a similar trend as those of the starter period. Both diets with an MCR of 50:50 (diet C and F) yielded superior FBW and BWG without significant differences between them. In addition, as in the starter period, there was no observed significant difference to diet D (HM 55:45) for FBW and BWG, but a trend was seen. However, in contrast to the starter period, the FCR between diet C and F was significantly different. The FCR of the SBM based diet F was significantly impaired. The decline of the MCR (diet A) led to significantly impaired DMI and related zoo-technical results. DMI with diet F was the highest (*p* < 0.05), but not significantly different from diet C.

Summarizing the results of the total growth period underlines that diet C (HM 50:50) and diet F (SBM 50:50) achieved superior zoo-technical results, that were not significantly different from diet D (HM 55:45). However, birds fed diet F also revealed a significantly higher DMI, leading to an impaired FCR, even lower than that of diet A (HM 40:60). Diet A also led to the lowest DMI and declined FBW and BWG (*p* < 0.05).

### 3.2. Apparent Precaecal Digestibility

Both the apD of crude protein and all AA, except for Met, were not significantly different between the HM diets A–E (Table 5). The apD of Met was the highest in diet E (HM 60:40) and significantly different (*p* < 0.05) from both diet A (HM 40:60) and diet F (SBM 50:50). A comparison of diet C (HM 50:50) and F (SBM 50:50) showed no significant differences in the apD of crude protein and individual AAs. However, significant differences (*p* < 0.05) in the apD of Met, Thr, and Leu between diet E (HM 60:40) and F (SBM 50:50) were observed. ApD of Arg and Val differed significantly (*p* < 0.05) between diet D (HM 55:45), E (HM 60:40), and F (SBM 50:50), while apD of Cys was even significantly lower (*p* < 0.05) in diet F than in all HM diets, except for diet C (HM 50:50).

### 3.3. Protein Quality and Met Efficiency

In the starter period (Table 6), the N deposition (*ND*) data and derived parameters of both dietary protein quality (*b*, NPU_std._) and Met efficiency (*bc^−^*^1^) are in general agreement with the summarized results of the growth study (Table 5). Diet C (HM 50:50) and diet F (SBM 50:50) yielded the highest *ND* data, and diet A (HM 40:60) was inferior (*p* < 0.05). Increasing the MCR led to a significant increase in *ND* values yielding a plateau with diet C. As a consequence, a further increase of the MCR tended to decrease *ND*. The protein quality parameters of diet C (HM 50:50) were neither significantly different from diet F (SBM 50:50), diet D (HM 55:45), nor diet E (60:40). However, diet A (HM 40:60) yielded the lowest protein quality (*p* < 0.05). The observed Met efficiency was the highest across diets A (HM 40:60), B (HM 55:45), and C (HM 50:50), but declined (*p* < 0.05) when the MCR increased (diets D,E). Diet F (SBM 50:50) tended to have a lower Met efficiency as compared to diets A, B, and C (*p* > 0.05).

In the grower period, diet C (HM 50:50) and F (SBM 50:50) yielded the highest *ND* data, but differed only numerically from diet B (HM 45:55) and D (HM 55:45). Diets A (HM 40:60) and E (HM 60:40) had the lowest *ND* values. Diet C (HM 50:50) yielded superior protein quality, but the value was not significantly different from diets B (HM 45:55), D (HM 55:45), and F (SBM 50:50). The lowest protein quality was observed with diets A (HM 40:60) and E (HM 60:40). Surprisingly, the protein quality parameters of diet F (SBM 50:50) were not significantly different from diet A (HM 40:60). As in the starter period, Met efficiency was the highest in diets with a MCR < 1 (diets A and B), but only numerically different from diet C (HM 50:50). However, Met efficiency in diet F (SBM 50:50) was significantly lower compared to diets A and B. Diet E (HM 60:40) generally impaired the Met efficiency (*p* < 0.05).

In summary across the entire growth period, *ND* data between diet C (HM 50:50) and diet F (SBM 50:50) were very similar (*p* > 0.05) and differed only numerically from diet B. In addition, no significant effects on *ND* were observed between diets B (HM 45:55), D (HM 55:45), and E (HM 60:40). As in the starter and grower period, diet A (HM 40:60) achieved both inferior *ND* data and dietary protein quality (*p* < 0.05). Increasing the MCR above 40:60 improved the dietary protein quality significantly, but the Met efficiency remained more or less unchanged up to an MCR = 50:50, as in diet C. A further increase of the MCR led to a decline (*p* < 0.05) in the Met efficiency with diets D and E. An intermediate Met efficiency was observed with the SBM based diet F, but not significantly below the superior level of diet C with an equal MCR. 

### 3.4. Body Protein and Body Fat Deposition

The graded dietary MCR was also a factor of influence on the nutrient deposition in the bird’s bodies (Table 7). In the starter period, diet A (HM 40:60) yielded both inferior crude protein (CPD) and crude lipid deposition (CLD). Both parameters could be significantly improved by increasing the MCR to 50:50 (diet C), while a further increase of the MCR had no significant effect. CPD was highest in diet F (SBM 50:50), but not significantly different from diet C (HM 50:50) and D (HM 55:45). In contrast, the highest CLD was observed in diets C and D. Both values were significantly different from CLD of diets A and D and the reference diet F.

Accordingly, in the grower period inferior CPD and CLD were observed in diet A (HM 40:60), but could be improved by increasing the MCR to 50:50 (diet C). In contrast to the starter period, a further increase of the MCR led to a significant decrease of CPD and CLD in diets D and E. Again, CPD was highest in diet F (SBM 50:50), but not significantly different from diet C (HM 50:50). The highest CLD was found in diet C with a significant difference to diet F. The observed results and trends of the grower period were confirmed in the total growth period.

## 4. Discussion

In accordance with recent studies [41,46,47], our current results underline the possibility of a complete substitution of SBM by partly defatted HM meal in diets for growing chickens. This is an important additional result, but not the main focus of this research to be discussed further.

Within each of the growth periods under study, the MCR of 50:50 led to superior results for the growth parameters, and therefore seems to be favorable in diets for growing broiler chickens. This confirms the observations of Moran Jr. [69], Baker et al. [8], Powell et al. [29], and Khan et al. [30]. However, an increased Met proportion of 55% did not impair growth. This agrees with Grau and Almquist [70] who stated that Met should contribute 50–55% of the total SAA content in diets for growing chickens. According to earlier observations [13,16,17,18,71,72,73,74], our results provide evidence that a Met ratio of less than 50% of the SAA impaired feed intake and growth response, respectively. In two studies Dilger and Baker [15,16] concluded explicitly that the elevated MCR caused an anorexic effect, and due to the fact that the experimental diets only differed in MCR, our results are in line with these conclusions. Sell et al. [72] assumed that an AA imbalance was the reason for the impaired feed intake and growth at high Cys proportions. Dilger and Baker [16] came to the same conclusion and stated that the growth-depressive effect of Cys could be already alleviated by the supplementation of small amounts of Met. A negative effect of an AA imbalance on feed intake was also observed by Kumar et al. [75]. Featherson and Rogler [18] suspected that an intestinal antagonism between Met and Cys causes the inferior growth at high Cys proportions. Both Sell et al. [76] and Lerner and Taylor [77] found evidence for an inhibition of intestinal Met absorption with increasing Cys supply. In contrast, Graber et al. [13], Sünder et al. [33], and Pesti et al. [78] stated that Cys could supply >50% of the total SAA requirement. Ohta and Ishibashi [79] observed a maximal growth response at an MCR of 45:55, but also concluded that the optimal Cys ratio increases with increasing SAA levels in the diets.

Because growth parameters in our study could be improved by increasing the MCR up to 50:50, it can be concluded that the birds´ requirement for Cys was efficiently covered and metabolic Met degradation in order to provide Cys did not exceed the physiological normal level. 

The MCR of 60:40 in the present study led to a decline in feed intake and growth compared to a MCR of 50:50. This observation was even significant in the grower period and could be explained by a metabolic Cys deficiency resulting in elevated Met degradation and lower Met supply for growth. In contrast, Featherston and Rogler [18] and Grau and Almquist [70] stated that a MCR of 76:24 was superior for growth in diets with an adequate SAA supply. Marquardt and Campbell [74] and Beck et al. [80] also found superior growth performance of broiler chickens at a Met proportion ≥60% of the SAA. However, several other authors also found an age-dependent effect on the optimal MCR. For example, Wheeler and Latshaw [31] observed a decline in the optimal Cys ratio from 54% (1–21 days) to 38–43% (21–42 days) of the SAA due to increasing importance of Met for growth in older birds. The NRC [28] also recommended an increasing Cys ratio, 43% of the SAA in the first three weeks of age and subsequently 47% of the SAA. In contrast, Kalinowski et al. [81,82] did not observe a different optimal Cys ratio in growing chickens between 0–3 and 3–6 weeks of age (44% resp. 47% of the SAA, but dependent on genotype).

The FCR in our study tended to be improved in diets with an MCR of 50:50 in comparison to a MCR of 60:40. Moran Jr. [69] found a significantly improved FCR when increasing the Cys ratio from 40 to 50% of the SAA in diets for broiler chickens from 0–14 days of age. However, Featherston and Rogler [18] observed a significantly improved FCR in diets with an MCR of 61:39, compared to diets with an MCR of 44:56. Similar results were reported elsewhere [16,31]. In contrast, Graber et al. [13] found no significant effects on the FCR when the Cys proportion was between 0 and 58% of the SAA.

Nevertheless, it has to be kept in mind that in contrast to our experiment, in most of the reported studies the sum of SAA in the diets was not held constant. In addition, the SAA were not generally in a limiting position. However, Sasse and Baker [71] emphasized that if the total SAA content exceeds the requirement, the optimal Cys proportion will be overestimated. 

A direct comparison of diet C (HM 50:50) and diet F (SBM 50:50) yielded no significant differences in growth parameters indicating the possibility of a complete substitution of SBM by HM. This observation is in line with the results of Neumann et al. [46] and Leiber et al. [50]. However, the FCR with diet F was slightly impaired as related to actual results with a similar ingredient composition [46,47,48], but in these studies a more balanced SAA:Lys ratio was applied. As mentioned above, the limiting position of SAA was intentional in our current study. On the contrary, Dabbou et al. [45] found a significantly impaired FCR in AA-balanced diets with 15% HM compared to a control diet with SBM. Leiber et al. [50] applied 7.8% HM in diets for growing chickens and observed no significant effect on FCR.

The observed results of apD of CP and selected AAs showed no significant differences between diet C (HM 50:50) and F (SBM 50:50) and are in line with findings of Velten et al. [49]. The MCR did not have a consistent effect on AA digestibility. The significant response of increased apD with a higher MCR between diet A (HM 40:60) and diet E (HM 60:40) should not be over interpreted according to the methodical background. Increasing Met concentration in the feed protein yielded elevated apD of Met due to the well-known relationship between the level of AA intake and observed apD. Additionally, this observed effect is supported by the elevated contents of crystalline DL-Met, which are expected to be completely digestible in the digestive tract. Several authors examined the coefficients of apparent digestibility of the total intestinal tract (CITAD) and apparent ileal digestibility coefficients (AIDC) for HM [43,44]. De Marco et al. [43] observed a CITAD for CP of 0.51, but the AIDC differed between e.g., 0.42 (Lys), 0.46 (Met) and 0.82 (Cys). Shiavone et al. [44] compared partly and highly defatted HM and found no effect on CITAD for CP (0.62) and the AIDC for Lys (0.80). However, AIDC for Met was higher in partly defatted HM (0.83 vs. 0.78), but the AIDC for Cys was higher in highly defatted HM (0.65 vs. 0.44). Nevertheless, different methodology and the fact that we examined the apD only in the mixed diets limit direct comparisons of the results. 

The dietary protein quality as reflected by the N intake independent model parameter *b* was significantly affected by the MCR. Diet C, with an MCR of 50:50, yielded a superior response indicating that this ratio supported the highest feed protein quality. Otherwise, an MCR of 40:60 led to a severe depression in the feed protein quality parameter indicating that a Met deficiency has an enormous impact on protein utilization. The fact that dietary protein quality was increased by elevating the MCR up to 50:50 validated that Met was the limiting AA. Regarding different age periods, both in the grower period and across the total growth period a MCR above 50:50 impaired the dietary protein quality. This effect was significant between diets C (HM 50:50) and E (HM 60:40) within these age periods, but only numerical differences were observed in the starter period. As a consequence, an age dependent effect on the optimal MCR is indicated and supports the increasing importance of Cys supply within the total SAA in older birds. In contrast to the actual results, Liebert et al. [83] observed superior protein quality with an MCR of 40:60 compared to an MCR of 51:49 in the grower period, but applied a corn-SBM-legume diets. Khan et al. [30] found no significant difference in protein quality and Met efficiency for a MCR between 46:54 and 51:49 in starter and grower period, but it was not designed as a systematic study with clearly graded MCR in the diet. In our study, we observed significantly lower protein quality for an MCR = 45:55 compared to an MCR = 50:50 in the starter and total growth period. However, the observed Met efficiency was not significantly influenced indicating that the effect on dietary protein quality was mostly related to the different Met concentration in the feed protein.

The dietary Met efficiency (*bc*^−1^) provides at least a measure for the quantity of Met that is needed to yield one unit of *ND*, but also indirectly indicates how the individual AA is involved in metabolic processes [61]. As a consequence, the daily quantity of an individual AA for metabolic needs strongly influences the numerical value of *bc*^−1^ for the AA under study. However, comparisons between *bc*^−1^ data for the limiting AA under study yield important information about the level of utilization for the metabolic purposes taking into account both the maintenance and performance processes. Due to the identical diet composition and the fact that Met was the limiting AA in all diets, comparison of Met efficiency data (*bc*^−1^) can be applied as an important tool to identify metabolic situations with different efficiencies of Met utilization. As observed, *bc*^−1^ was highest in the diets A (HM 40:60), B (HM 45:55), and C (HM 50:50) with an MCR of 50:50 or lower. Farke et al. [32] also stated that an MCR of 40:60 improves the dietary Met-efficiency due to minimized Met degradation in diets with a low Met proportion. In our study, increasing the MCR in diets D and E impaired the Met efficiency significantly, indicating that Met was partially degraded to improve the metabolic Cys supply. Otherwise, results with diets A, B, and C underline that the MCR was sufficient and Met catabolism to yield Cys was not stimulated. These results are in line with Khan et al. [30] who also found no differences in Met efficiency between MCRs of 46:54, 48:52, and 51:49. 

Directly comparing the HM diet C (50:50) and the SBM diet F (50:50) over the age periods under study provided no significant effect both on protein quality and Met efficiency. However, as in accord with reports from Neumann et al. [46,47] and Velten et al. [49], diet C (HM 50:50) yielded a trend of superior protein quality and Met efficiency as compared to the SBM based diet F. This trend might indicate an improved AA availability in the animal protein-based diet (HM 50:50) as compared to the plant based diet (SBM 50:50).

Both CPD and CLD increased with an increasing MCR ratio up to 50:50 and decreased with a ratio of 55:45 or higher. A low MCR as applied in diet A (HM 40:60) created a stronger negative effect than a lower Cys percentage of SAA as utilized in diet E (HM 60:40). Accordingly, Fatufe and Rodehutscord [84] also observed an increase in CLD with increasing Met concentration in diets for growing chickens, but they observed no effect on CPD. Otherwise, Graber et al. [13] did not find an effect of the MCR on the body composition of growing chickens. However, in contrast to our study, the total SAA content was not held constant in those investigations. 

The observed effects on CPD and CLD in the bird´s bodies between diet C (HM 50:50) and F (SBM 50:50) were expected and can be explained by the different energy content between control and insect-meal-based experimental diets. Both the higher fat content in the insect meal and the lower proportion of wheat and maize in the SBM based diet F created this response founded on the energy content. However, it was not the aim within the current study to compensate this difference, but to demonstrate by example the effect that a high inclusion rate of insect meal could have on body composition when the dietary energy content is not adapted. Generally, this effect on dietary energy concentration can be alleviated by diet formulation with a modulated oil percentage.

## 5. Conclusions

In conclusion, the partly defatted HM meal under study could completely substitute SBM in diets for growing chickens without significant effects on growth, apD, and dietary protein quality when adequately supplemented with SAA. The results from chicken diets with a suboptimal SAA supply and Met as limiting AA indicated that an optimal dietary MCR of 50:50 was also valid at high inclusion rates of insect meal. However, a decline of the MCR to 40:60 impaired growth and feed efficiency. Otherwise, an elevated MCR exceeding the optimal ratio was not so meaningful due to the possibility of Met degradation in order to ensure the metabolic Cys supply. The observations underline the importance of taking into account not only quantitative requirements but also the optimal balance between dietary amino acids, namely the SAA methionine and cysteine. The high inclusion rate of HM and its specific AA composition did not modify the optimal MCR in broiler chicken diets.

## Figures and Tables

**Table 1:** Analyzed amino acid content of the main feed ingredients (g/16 gN).

Amino Acid (g/16 gN)	Wheat	Maize	Soybean Meal	Partly Defatted *Hermetia illucens* Meal
Lys	2.72	2.62	6.07	5.42
Met	1.46	1.77	1.28	1.24
Cys	2.23	1.96	1.46	0.80
Thr	2.86	3.34	3.78	3.57
Arg	4.47	4.09	7.19	4.12
Val	3.85	4.34	4.37	5.36
Leu	6.18	11.37	7.32	6.24
Ile	3.07	3.18	4.34	3.86
His	2.19	2.65	2.53	2.73

**Table 2:** Composition of the experimental diets (g/kg).

	Starter Period (Days 1–21)						Grower Period (Days 22–35)					
Diet	A	B	C	D	E	F	A	B	C	D	E	F
	HM	HM	HM	HM	HM	SBM	HM	HM	HM	HM	HM	SBM
Met:Cys	40:60	45:55	50:50	55:45	60:40	50:50	40:60	45:55	50:50	55:45	60:40	50:50
*Hermetia* meal	230	230	230	230	230	-	212.8	212.8	212.8	212.8	212.8	-
Soybean meal	-	-	-	-	-	350	-	-	-	-	-	323.8
Wheat	500	500	500	500	500	398	462.5	462.5	462.5	462.5	462.5	368.2
Maize	140	140	140	140	140	112	129.5	129.5	129.5	129.5	129.5	103.6
Wheat starch	12.0	12.2	12.3	12.5	12.6	16.7	86.4	86.6	86.7	86.8	87.0	83.2
Soybean oil	70	70	70	70	70	75	65	65	65	65	65	80
DCP	10	10	10	10	10	16	8	8	8	8	8	12
CaCO_3_	11	11	11	11	11	11	8	8	8	8	8	8
NaCl	2	2	2	2	2	5	1	1	1	1	1	2.5
Premix ^1^	10	10	10	10	10	10	10	10	10	10	10	10
L-Lysine·HCl	5.65	5.65	5.65	5.65	5.65	3.74	5.21	5.21	5.21	5.21	5.21	3.44
L-Cysteine·HCl·H_2_O	2.24	1.73	1.22	0.73	0.22	0.03	2.06	1.58	1.13	0.66	0.19	0.03
L-Arginine	5.77	5.77	5.77	5.77	5.77	1.02	5.35	5.35	5.35	5.35	5.35	0.96
L-Threonine	1.35	1.35	1.35	1.35	1.35	0.85	1.23	1.23	1.23	1.23	1.23	0.77
DL-Methionine	-	0.35	0.71	1.04	1.39	0.65	-	0.33	0.65	0.96	1.29	0.59
TiO_2_	-	-	-	-	-	-	3	3	3	3	3	3

^1^ Added per kg of final diet: 2.1 g calcium, 0.8 g sodium, 5000 IU vitamin A, 1000 IU vitamin D3, 30 mg vitamin E, 2.6 mg vitamin B1, 4.8 mg vitamin B2, 3.2 mg vitamin B6, 20 μg vitamin B12, 3 mg vitamin K3, 50 mg nicotinic acid, 10 mg calcium pantothenate, 0.9 mg folic acid, 100 μg biotin, 1000 mg choline chloride, 50 mg Fe as iron-II-sulfate, monohydrate, 15 mg Cu as copper-II-sulfate, pentahydrate, 120 mg Mn as manganese-II-oxide, 70 mg Zn as zinc oxide, 1.4 mg I as calcium iodate, hexahydrate, 0.28 mg Se as sodium selenite, 0.55 mg Co as alkaline cobalt-II-carbonate, monohydrate, and 100 mg butylhydroxytoluol.

**Table 3:** Analyzed nutrient content of the experimental diets.

	Starter Period (Days 1–21)						Grower Period (Days 22–35)					
Diet	A	B	C	D	E	F	A	B	C	D	E	F
	HM	HM	HM	HM	HM	SBM	HM	HM	HM	HM	HM	SBM
Met:Cys	40:60	45:55	50:50	55:45	60:40	50:50	40:60	45:55	50:50	55:45	60:40	50:50
Crude nutrient composition (% DM)												
Crude protein	23.8	23.9	23.4	23.3	23.3	22.9	22.2	22.0	22.1	22.5	21.6	21.1
Crude lipids	12.1	12.8	12.8	12.8	12.2	10.7	11.4	11.1	11.1	11.4	11.5	11.0
Crude fiber	4.3	4.2	3.9	3.9	3.8	4.3	3.7	3.7	3.7	3.7	3.8	4.0
Crude ash	5.5	5.7	5.6	5.5	5.6	7.2	5.2	5.1	5.2	5.2	5.2	6.2
AME_n_ (MJ/kg DM) ^1^	15.8	15.8	15.8	15.8	15.8	14.2	15.8	15.8	15.8	15.8	15.8	14.5
Amino acid composition (g/16 gN) ^2^												
Lys	6.16	6.16	6.16	6.16	6.16	6.41	6.16	6.16	6.17	6.17	6.17	6.42
Met	1.22	1.38	1.54	1.69	1.85	1.61	1.23	1.39	1.54	1.70	1.90	1.61
Met + Cys	3.08	3.08	3.08	3.08	3.08	3.21	3.08	3.08	3.09	3.08	3.08	3.21
Thr	3.70	3.70	3.70	3.70	3.70	3.85	3.70	3.70	3.70	3.70	3.70	3.85
Arg	6.47	6.47	6.47	6.47	6.47	6.73	6.47	6.47	6.47	6.47	6.47	6.73
Val	4.49	4.49	4.49	4.49	4.49	4.13	4.49	4.49	4.49	4.49	4.49	4.13
Leu	6.00	6.00	6.00	6.00	6.00	7.04	6.00	6.00	6.00	6.00	6.00	7.04
Ile	3.31	3.31	3.31	3.31	3.31	3.89	3.31	3.31	3.31	3.31	3.31	3.89
His	2.36	2.37	2.37	2.37	2.37	2.39	2.37	2.37	2.37	2.37	2.37	2.39

^1^ N-corrected apparent metabolizable energy, calculated according to WPSA [53]; ^2^ Final AA content of the mixed diets derived from analyzed AA composition of the ingredients.

**Table 4:** Results of growth and feed efficiency parameters ^1^.

Diet Met:Cys	A HM 40:60	B HM 45:55	C HM 50:50	D HM 55:45	E HM 60:40	F SBM 50:50
Starter period (days 1–21)						
FBW (g)	622 ^a^ ± 91	847 ^b^ ± 72	978 ^cd^ ± 83	911 ^bcd^ ± 78	879 ^bc^ ± 72	1021 ^d^ ± 46
BWG (g/d)	27 ^a^ ± 4	38 ^b^ ± 3	44 ^cd^ ± 4	41 ^bcd^ ± 4	39 ^bc^ ± 3	46 ^d^ ± 2
DMI (g/d)	40.4 ^a^ ± 4.8	51.1 ^b^ ± 4.6	54.4 ^b^ ± 4.3	51.7 ^b^ ± 2.4	49.9 ^b^ ± 5.0	61.7 ^c^ ± 4.6
FCR	1.51 ^a^ ± 0.15	1.36 ^ab^ ± 0.07	1.24 ^c^ ± 0.07	1.27 ^bc^ ± 0.08	1.27 ^bc^ ± 0.09	1.34 ^abc^ ± 0.06
Grower period (days 22–35)						
FBW (g)	1307 ^a^ ± 227	1780 ^b^ ± 150	2064 ^c^ ± 232	1838 ^bc^ ± 153	1628 ^b^ ± 161	2108 ^c^ ± 150
BWG (g/d)	49 ^a^ ± 11	67 ^bc^ ± 10	78 ^c^ ± 13	66 ^bc^ ± 8	54 ^ab^ ± 7	78 ^c^ ± 8
DMI (g/d)	77.5 ^a^ ± 16.3	99.4 ^bc^ ± 12.7	114.1 ^cd^ ± 14.2	102.5 ^bc^ ± 10.2	86.3 ^ab^ ± 8.6	131.2 ^d^ ± 12.5
FCR	1.59 ^ab^ ± 0.11	1.51 ^b^ ± 0.17	1.49 ^b^ ± 0.12	1.55 ^ab^ ± 0.07	1.62 ^ab^ ± 0.09	1.69 ^a^ ± 0.03
Total growth period (days 1–35)						
FBW (g)	1307 ^a^ ± 227	1780 ^b^ ± 150	2064 ^c^ ± 232	1838 ^bc^ ± 153	1628 ^b^ ± 161	2108 ^c^ ± 150
BWG (g/d)	36 ^a^ ± 7	49 ^b^ ± 4	57 ^c^ ± 7	51 ^bc^ ± 4	45 ^b^ ± 5	59 ^c^ ± 4
DMI (g/d)	55.6 ^a^ ± 9.0	70.5 ^bc^ ± 6.2	78.3 ^c^ ± 7.1	72.0 ^bc^ ± 8.1	64.5 ^ab^ ± 5.8	89.5 ^d^ ± 7.2
FCR	1.55 ^ab^ ± 0.08	1.43 ^abc^ ± 0.10	1.37 ^c^ ± 0.08	1.42 ^c^ ± 0.03	1.44 ^bc^ ± 0.06	1.53 ^a^ ± 0.03

^1^ Means ± standard deviations, different superscript letters reveal significant differences between diets (*p* < 0.05). FBW = Final body weight, BWG = Body weight gain, DMI = Dry matter intake, and FCR = Feed conversion rate (g DMI/g BWG).

**Table 5:** Apparent precaecal digestibility (%) of crude protein (CP) and selected amino acids in the experimental diets (*n* = 5) ^1^.

Diet Met:Cys	A HM 40:60	B HM 45:55	C HM 50:50	D HM 55:45	E HM 60:40	F SBM 50:50
CP	74.2 ± 4.0	76.7 ± 2.8	76.0 ± 2.9	79.2 ± 2.7	79.6 ± 2.0	77.7 ± 1.9
Lys	83.4 ± 2.2	84.9 ± 3.4	83.4 ± 3.9	86.7 ± 2.5	88.1 ± 1.6	83.4 ± 2.0
Met	82.1 ^a^ ± 2.2	85.8 ^ab^ ± 4.5	87.6 ^ab^ ± 5.4	88.3 ^ab^ ± 2.6	91.3 ^b^ ± 1.6	83.6 ^a^ ± 2.2
Cys	66.6 ^b^ ± 4.0	68.2 ^b^ ± 3.6	61.6 ^ab^ ± 4.1	69.8 ^b^ ± 4.8	63.3 ^b^ ± 6.1	52.7 ^a^ ± 1.0
Thr	73.2 ^ab^ ± 2.2	76.2 ^ab^ ± 4.3	72.9 ^ab^ ± 4.1	77.7 ^ab^ ± 3.0	78.8 ^b^ ± 2.6	69.5 ^a^ ± 5.7
Arg	88.4 ^ab^ ± 1.7	89.0 ^ab^ ± 2.5	87.7 ^ab^ ± 3.0	89.9 ^b^ ± 2.2	91.3 ^b^ ± 1.2	84.9 ^a^ ± 1.4
His	79.9 ± 2.2	81.2 ± 2.9	80.4 ± 3.2	83.1 ± 2.7	84.3 ± 1.7	81.3 ± 1.7
Ileu	80.9 ± 1.9	82.3 ± 3.8	81.0 ± 4.2	83.9 ± 2.9	85.4 ± 2.2	79.3 ± 2.0
Leu	81.7 ^ab^ ± 2.2	83.3 ^ab^ ± 4.6	81.7 ^ab^ ± 4.0	84.8 ^ab^ ± 2.8	86.3 ^b^ ± 1.9	79.7 ^a^ ± 2.1
Phe	83.3 ± 1.8	85.0 ± 3.9	82.8 ± 3.7	86.3 ± 2.6	84.4 ± 1.9	82.0 ± 1.6
Val	73.4 ^ab^ ± 2.7	76.2 ^ab^ ± 3.8	73.9 ^ab^ ± 3.2	78.7 ^b^ ± 2.5	79.6 ^b^ ± 2.8	71.5 ^a^ ± 2.2

^1^ Means ± standard deviations, different superscript letters reveal significant differences between diets (*p* < 0.05).

**Table 6:** Results of dietary protein quality (*b*, NPU_std._) and Met efficiency (*bc*^−1^) ^1^.

Diet Met:Cys	A HM 40:60	B HM 45:55	C HM 50:50	D HM 55:45	E HM 60:40	F SBM 50:50
Starter period (days 1–21)						
*ND* ^2^	1294 ^a^ ± 91	1511 ^b^ ± 81	1636 ^cd^ ± 58	1594 ^bc^ ± 57	1576 ^bc^ ± 56	1706 ^d^ ± 33
*b* (×10^6^) ^3^	140 ^a^ ± 15	157 ^b^ ± 9	178 ^c^ ± 11	174 ^bc^ ± 11	175 ^bc^ ± 13	175 ^c^ ± 8
NPU_std._ (%) ^3^	47.3 ^a^ ± 4.0	52.0 ^b^ ± 2.2	57.1 ^c^ ± 2.5	56.0 ^bc^ ± 2.7	56.2 ^bc^ ± 3.1	56.4 ^c^ ± 1.9
*bc*^−1^ (×10^6^) ^3^	114 ^bc^ ± 12	114 ^bc^ ± 6	116 ^c^ ± 7	103 ^ab^ ± 7	94 ^a^ ± 7	109 ^bc^ ± 5
Grower period (days 22–35)						
*ND* ^2^	1185 ^ab^ ± 155	1412 ^c^ ± 165	1532 ^c^ ± 170	1383 ^bc^ ± 122	1156 ^a^ ± 111	1519 ^c^ ± 100
*b* (×10^6^) ^3^	189 ^a^ ± 16	222 ^bc^ ± 32	235 ^c^ ± 27	215 ^abc^ ± 12	195 ^ab^ ± 13	212 ^abc^ ± 7
NPU_std._ (%) ^3^	49.6 ^a^ ± 3.1	55.5 ^bc^ ± 5.5	57.9 ^c^ ± 4.4	54.4 ^abc^ ± 2.3	50.8 ^ab^ ± 2.5	54.0 ^abc^ ± 1.3
*bc*^−1^ (×10^6^) ^3^	154 ^d^ ± 13	160 ^d^ ± 23	153 ^cd^ ± 18	127 ^b^ ± 7	106 ^a^ ± 7	132 ^bc^ ± 4
Total growth period (days 1–35)						
*ND* ^2^	1250 ^a^ ± 101	1461 ^b^ ± 60	1594 ^cd^ ± 83	1509 ^bc^ ± 57	1408 ^b^ ± 65	1631 ^d^ ± 55
*b* (×10^6^) ^3^	160 ^a^ ± 11	183 ^b^ ± 12	203 ^c^ ± 14	193 ^bc^ ± 7	187 ^b^ ± 9	193 ^bc^ ± 6
NPU_std._ (%) ^3^	48.0 ^a^ ± 2.5	53.1 ^b^ ± 2.6	57.3 ^c^ ± 2.7	55.3 ^bc^ ± 1.4	54.1 ^b^ ± 2.0	55.3 ^bc^ ± 1.1
*bc*^−1^ (×10^6^) ^3^	131 ^c^ ± 9	132 ^c^ ± 9	132 ^c^ ± 9	114 ^b^ ± 4	101 ^a^ ± 5	120 ^c^ ± 3

^1^ Means ± standard deviations, different superscript letters reveal significant differences between diets (*p* < 0.05). ^2^ *ND* = Daily nitrogen deposition (mg/BW_kg_^0.67^). ^3^ See Equations (4)–(6).

**Table 7:** Body crude protein (CPD) and body crude fat deposition (CLD) in the growth periods ^1^.

Diet Met:Cys	A HM 40:60	B HM 45:55	C HM 50:50	D HM 55:45	E HM 60:40	F SBM 50:50
Starter period (days 1–21)						
CPD (g/d)	3.93 ^a^ ± 0.63	5.54 ^b^ ± 0.50	6.58 ^cd^ ± 0.59	6.12 ^d^ ± 0.56	5.92 ^bc^ ± 0.52	7.04 ^d^ ± 0.33
CLD (g/d)	3.22 ^a^ ± 0.49	5.25 ^bc^ ± 0.46	6.39 ^d^ ± 0.55	6.01 ^d^ ± 0.52	5.88 ^cd^ ± 0.49	4.99 ^b^ ± 0.23
Grower period (days 22–35)						
CPD (g/d)	7.28 ^a^ ± 1.63	10.61 ^bc^ ± 1.54	12.73 ^cd^ ± 2.07	10.71 ^bc^ ± 1.25	8.43 ^ab^ ± 1.18	12.83 ^d^ ± 1.32
CLD (g/d)	8.33 ^a^ ± 1.71	10.87 ^ab^ ± 1.54	13.51 ^c^ ± 2.17	11.47 ^bc^ ± 1.31	9.44 ^ab^ ± 1.26	11.36 ^ab^ ± 1.09
Total growth period (days 1–35)						
CPD (g/d)	5.56 ^a^ ± 1.00	7.82 ^b^ ± 0.68	9.24 ^cd^ ± 1.06	8.20 ^bc^ ± 0.70	7.22 ^b^ ± 0.73	9.87 ^d^ ± 0.72
CLD (g/d)	5.45 ^a^ ± 0.96	7.96 ^b^ ± 0.68	9.77 ^c^ ± 1.11	8.69 ^b^ ± 0.73	7.63 ^b^ ± 0.76	7.85 ^b^ ± 0.56

^1^ Means ± standard deviations, different superscript letters reveal significant differences between diets (*p* < 0.05).
